# Associations between latent classes of left-behind experience and non-suicidal self-injury among rural adolescents: the sequential mediating roles of family functioning and shame

**DOI:** 10.3389/fpubh.2026.1964648

**Published:** 2026-10-14

**Authors:** Wei Mi, Shiying Tan, Zhanqun Gao, Qing Yang, Cuixiao Wang

**Affiliations:** 1 School of Nursing, Hunan University of Medicine, Huaihua, Hunan, China; 2 Hunan University of Medicine General Hospital, Huaihua, Hunan, China; 3 Psychological and Behavioral Medicine Key Research Base, Hunan University of Medicine, Huaihua, Hunan, China

**Keywords:** family functioning, latent class analysis, left-behind adolescents, non-suicidal self-injury, sequential mediation, shame

## Abstract

**Objective:**

This study aimed to identify latent classes of left-behind experience among rural Chinese adolescents and to examine the sequential mediating roles of family functioning and shame in the relationship between left-behind experience and NSSI.

**Methods:**

A cross-sectional survey was conducted among 1,735 rural middle school students with left-behind experience from two schools in Hunan Province, China, in 2024. Latent class analysis (LCA) was performed to identify subgroups of left-behind experience based on four indicators: age at parental separation, parental migration pattern, duration of parental migration, and frequency of communication with migrant parents. Correlation analysis, multiple linear regression, and a bootstrap-based sequential mediation model with 5,000 resamples were used to examine the relationships among latent class membership, family functioning, shame, and NSSI.

**Results:**

LCA identified three distinct latent classes of left-behind experience: a low-risk group, a moderate-risk group, and a high-risk group. Correlation analyses showed that latent class membership was significantly associated with family functioning (*ρ* = −0.149, *p* < 0.05), shame (*ρ* = 0.139, *p* < 0.001), and NSSI (*ρ* = 0.142, *p* < 0.05). Family functioning was negatively associated with shame (*r* = −0.132, *p* < 0.001) and NSSI (*r* = −0.235, *p* < 0.001), whereas shame was positively associated with NSSI (*r* = 0.256, *p* < 0.001). Multiple regression analyses indicated that family functioning was negatively associated with shame (*β* = −0.126, *p* < 0.001) and NSSI (*β* = −0.204, *p* < 0.001), while shame was positively associated with NSSI (*β* = 0.229, *p* < 0.001). Sequential mediation analyses further indicated that, compared with the high-risk group, both the low-risk group (*B* = −0.107, 95% CI: −0.243 to −0.016) and the moderate-risk group (*B* = −0.143, 95% CI: −0.306 to −0.027) were associated with lower NSSI levels through the sequential pathway involving family functioning and shame.

**Conclusion:**

Left-behind experience among rural adolescents is heterogeneous, and different patterns are associated with varying levels of NSSI risk. Significant indirect associations involving family functioning and shame were observed between left-behind experience and NSSI, including a sequential indirect association through family functioning and shame. These findings suggest that family functioning and shame may represent relevant psychosocial factors to consider in future prevention efforts targeting NSSI among high-risk left-behind adolescents.

## 1 Introduction

Non-suicidal self-injury (NSSI) refers to the deliberate destruction of one’s own body tissue without suicidal intent and has emerged as a major public health concern among adolescents worldwide (1). Common forms of NSSI include cutting, scratching, burning, and self-hitting. Although NSSI is performed without suicidal intent, it is strongly associated with depression, anxiety, emotional dysregulation, suicidal ideation, and suicide attempts, thereby imposing substantial burdens on adolescents’ psychological well-being and social functioning (1–3). Recent evidence indicates that the prevalence of NSSI among Chinese adolescents remains alarmingly high, particularly among vulnerable populations residing in rural areas (4).

With rapid urbanization and labor migration in China, a large number of rural children and adolescents have experienced parental migration. These individuals, commonly referred to as left-behind children (LBC), remain in rural communities while one or both parents migrate to urban areas for employment (5). Long-term parental absence may reduce emotional support, parental supervision, and family interaction, increasing the risk of emotional and behavioral problems (6). Previous studies have consistently reported that left-behind adolescents are more likely to experience depression, loneliness, anxiety, self-harm, and NSSI than their non-left-behind counterparts (4, 7, 8).

Despite extensive research on left-behind adolescents, most previous studies have conceptualized left-behind experience as a dichotomous variable (left-behind vs. non-left-behind) (9). Such an approach may obscure substantial heterogeneity in parental migration experiences. In reality, left-behind adolescents differ considerably in age at parental separation, migration duration, migration pattern (father-only, mother-only, or both-parent migration), and frequency of parent–child communication. These characteristics may contribute to distinct developmental trajectories and psychological outcomes. Latent class analysis (LCA) is a person-centered statistical approach that enables the identification of unobserved subgroups based on multiple indicators simultaneously (10). However, few studies have employed LCA to characterize heterogeneous patterns of left-behind experiences and their associations with NSSI.

Family functioning represents one of the most important protective factors for adolescent mental health. According to family systems theory, healthy family functioning provides emotional warmth, effective communication, adaptability, and social support, thereby buffering the negative consequences of adverse life experiences (11). Conversely, poor family functioning has been associated with emotional dysregulation, psychological distress, and self-injurious behaviors among adolescents (12, 13). For left-behind adolescents, prolonged parental separation may weaken family cohesion and communication, resulting in impaired family functioning and increased vulnerability to NSSI.

Another important factor associated with NSSI is shame. Shame is a self-conscious emotion characterized by negative self-evaluation, feelings of inferiority, self-criticism, and social withdrawal (14). Previous studies have demonstrated that adolescents with elevated shame are more likely to engage in NSSI as a maladaptive emotion-regulation strategy or form of self-punishment (15, 16). Furthermore, adverse family environments may exacerbate shame by reducing emotional support and increasing negative self-perceptions (17).

Importantly, family functioning and shame may operate sequentially rather than independently. Poor family functioning may undermine adolescents’ emotional security and self-worth, thereby increasing shame and ultimately contributing to NSSI. Although previous studies have investigated individual mediating mechanisms linking adverse childhood experiences to NSSI, evidence regarding the sequential mediating roles of family functioning and shame remains limited, particularly among left-behind adolescents.

Therefore, the present study aimed to: (1) identify latent classes of left-behind experiences among rural Chinese adolescents using LCA; (2) examine differences in NSSI across latent classes; and (3) investigate whether family functioning and shame sequentially mediate the association between left-behind experience patterns and NSSI. We hypothesized that adolescents belonging to higher-risk left-behind classes would report poorer family functioning, higher levels of shame, and more severe NSSI, and that family functioning and shame would jointly mediate this relationship.

## 2 Methods

### 2.1 Study design and participants

A cross-sectional survey was conducted between March and June 2024 in two rural secondary schools in Hunan Province, China. Cluster sampling was used to recruit participants. The inclusion criteria were: (1) students enrolled in Grades 7–12; (2) having experienced parental migration during childhood or adolescence; and (3) ability to complete the questionnaire independently. Students with severe missing data or who declined participation were excluded. A total of 1,735 adolescents with left-behind experience were included in the final analysis. The study was approved by the Ethics Committee of Hunan University of Medicine [Approval No. 2025(H03009)]. Written informed consent was obtained from participants and their legal guardians before data collection.

### 2.2 Measures

#### 2.2.1 Sociodemographic characteristics

A self-administered questionnaire was used to collect demographic information, including sex, age, grade, only-child status, annual household income, parental occupation, parental educational level, and left-behind-related characteristics.

#### 2.2.2 Left-behind experience indicators

For the present study, left-behind experience was operationally defined as having experienced separation from one or both parents for at least 6 months because the parent(s) migrated away from the place of residence for employment. Adolescents who reported such an experience were considered eligible for the present analysis. Four indicators were used to characterize left-behind experiences:

1. Age at first parental separation;
2. Left-behind type (father-only migration, mother-only migration, or both-parent migration);
3. Duration of parental migration;
4. Frequency of communication with migrant parents.

These variables were entered into latent class analysis (LCA) to identify distinct patterns of left-behind experience.

#### 2.2.3 Non-suicidal self-injury

NSSI was assessed using the Adolescents Self-Harm Scale, revised by Feng (18) and subsequently modified by Yu et al. (8) for use among Chinese adolescents. The scale assesses 17 forms of self-injurious behavior occurring during the previous 12 months. Participants reported the frequency and physical severity of each behavior, which were used to derive a continuous NSSI severity score, with higher scores indicating greater NSSI severity. For the descriptive analysis in Table 1, participants reporting any NSSI behavior were classified as the NSSI group, whereas those reporting no NSSI behavior were classified as the non-NSSI group. The continuous NSSI severity score was used in the correlation, regression, and mediation analyses. Cronbach’s α in the present study was 0.85.

**Table 1:** Sociodemographic characteristics of participants according to NSSI status.

Variables	Overall (*N* = 1735)	NSSI (*n* = 846)	Non-NSSI (*n* = 889)	*χ*^2^/*t*	*p* value
Sex				14.487	<0.001
Male	878 (50.6)	388 (45.9)	490 (55.1)		
Female	857 (49.4)	458 (54.1)	399 (44.9)		
Age, Mean (SD)	14.76 (1.43)	14.50 (1.29)	15.01 (1.51)	−7.572	<0.001
Grade				79.83	<0.001
Grade 7	307 (17.7)	178 (21.0)	129 (14.5)		
Grade 8	481 (27.7)	268 (31.7)	213 (24.0)		
Grade 9	434 (25.0)	231 (27.3)	203 (22.8)		
Grade 10	258 (14.9)	95 (11.2)	163 (18.3)		
Grade 11	188 (10.8)	53 (6.3)	135 (15.2)		
Grade 12	67 (3.9)	21 (2.5)	46 (5.2)		
Only-child status				0.129	0.719
Yes	254 (14.6)	127 (15.0)	127 (14.3)		
No	1,481 (85.4)	719 (85.0)	762 (85.7)		
Annual household income (RMB)				16.942	0.001
<20,000	510 (29.4)	265 (31.3)	245 (27.6)		
20,000–50,000	632 (36.4)	331 (39.1)	301 (33.9)		
60,000–100,000	393 (22.7)	172 (20.3)	221 (24.9)		
>100,000	200 (11.5)	78 (9.2)	122 (13.7)		
Father’s occupation				2.734	0.603
Farmer	222 (12.8)	100 (11.8)	122 (13.7)		
Migrant worker	746 (43.0)	371 (43.9)	375 (42.2)		
Public servant	65 (3.7)	28 (3.3)	37 (4.2)		
Self-employed	233 (13.4)	112 (13.2)	121 (13.6)		
Other	469 (27.0)	235 (27.8)	234 (26.3)		
Mother’s occupation				6.083	0.193
Farmer	318 (18.3)	159 (18.8)	159 (17.9)		
Migrant worker	543 (31.3)	245 (29.0)	298 (33.5)		
Public servant	77 (4.4)	42 (5.0)	35 (3.9)		
Self-employed	240 (13.8)	113 (13.4)	127 (14.3)		
Other	557 (32.1)	287 (33.9)	270 (30.4)		
Father’s educational level				4.422	0.11
Junior high school or below	1,212 (69.9)	610 (72.1)	602 (67.7)		
Senior high school/technical school	432 (24.9)	192 (22.7)	240 (27.0)		
College degree or above	91 (5.2)	44 (5.2)	47 (5.3)		
Mother’s educational level				1.116	0.572
Junior high school or below	1,307 (75.3)	638 (75.4)	669 (75.3)		
Senior high school/technical school	371 (21.4)	184 (21.7)	187 (21.0)		
College degree or above	57 (3.3)	24 (2.8)	33 (3.7)		
Age at first parental separation				8.268	0.016
0–6 years	910 (52.4)	473 (55.9)	437 (49.2)		
7–12 years	600 (34.6)	275 (32.5)	325 (36.6)		
≥13 years	225 (13.0)	98 (11.6)	127 (14.3)		
Parental migration pattern				0.504	0.777
Father-only migration	541 (31.2)	258 (30.5)	283 (31.8)		
Mother-only migration	165 (9.5)	79 (9.3)	86 (9.7)		
Both-parent migration	1,029 (59.3)	509 (60.2)	520 (58.5)		
Duration of parental absence				0.504	0.777
<1 year	786 (45.3)	388 (45.9)	398 (44.8)		
1–3 years	441 (25.4)	217 (25.7)	224 (25.2)		
>3 years	508 (29.3)	241 (28.5)	267 (30.0)		
Frequency of parent–child communication				18.284	<0.001
Every 2 weeks	1,128 (65.0)	515 (60.9)	613 (69.0)		
Every 3–4 weeks	313 (18.0)	173 (20.4)	140 (15.7)		
Every 1–6 months	153 (8.8)	72 (8.5)	81 (9.1)		
Less than once every 6 months	141 (8.1)	86 (10.2)	55 (6.2)		
Primary caregiver during parental absence				0.167	0.92
One parent	535 (30.8)	257 (30.4)	278 (31.3)		
Grandparents	875 (50.4)	430 (50.8)	445 (50.1)		
Others	325 (18.7)	159 (18.8)	166 (18.7)		
Attitude toward parental migration				13.589	0.001
Supportive	1,035 (59.7)	469 (55.4)	566 (63.7)		
Opposed	87 (5.0)	52 (6.1)	35 (3.9)		
Neutral	613 (35.3)	325 (38.4)	288 (32.4)		
Shame score, mean (SD)	22.78 (15.54)	26.61 (15.72)	19.14 (14.46)	10.283	<0.001
Family functioning score, mean (SD)	13.52 (6.66)	12.00 (6.49)	14.98 (6.50)	−9.546	<0.001

#### 2.2.4 Family functioning

Family functioning was assessed using the six-item family functioning scale used by Cheng and Zou (19). The scale consists of six items assessing adolescents’ overall perceptions of family functioning. Each item is rated on a 5-point Likert scale, with higher total scores indicating better family functioning. The scale has demonstrated good reliability and validity among Chinese adolescents. Cronbach’s *α* in the present study was 0.94.

#### 2.2.5 Shame

Shame was measured using the 22-item Middle School Students’ Shame Scale developed by Qi et al. (20). The scale comprises four dimensions: behavioral shame, characterological shame, bodily shame, and ability-related shame. Items are rated on a 4-point Likert scale ranging from 0 (never) to 3 (frequently), with higher total scores indicating greater levels of shame. Cronbach’s *α* in the present study was 0.93.

### 2.3 Statistical analysis

All statistical analyses were performed using R version 4.5.1. Descriptive statistics were first calculated. Continuous variables are presented as means ± standard deviations (SD) and were compared between the NSSI and non-NSSI groups using *t*-tests, whereas categorical variables are presented as frequencies and percentages and were compared using Pearson’s chi-square tests. The four LCA indicators were entered as categorical variables using the following response categories: age at first parental separation (0–6 years, 7–12 years, and ≥13 years); parental migration pattern (father-only migration, mother-only migration, and both-parent migration); duration of parental absence (<1 year, 1–3 years, and >3 years); and frequency of parent–child communication (every 2 weeks, every 3–4 weeks, every 1–6 months, and less than once every 6 months). LCA was conducted using the poLCA package. Models containing one to six latent classes were estimated. Model selection was based on Akaike Information Criterion (AIC), Bayesian Information Criterion (BIC), adjusted BIC (aBIC), entropy values, class size, and interpretability. Lower AIC/BIC values indicate better model fit, whereas higher entropy values indicate greater classification accuracy. Spearman’s rank correlation and Pearson correlation analyses were subsequently performed to examine relationships among latent class membership, family functioning, shame, and NSSI. Multicollinearity was evaluated using variance inflation factors (VIFs). VIF values below 5 were considered acceptable. Hierarchical multiple regression analyses were then conducted. Family functioning, shame, and NSSI were entered sequentially as dependent variables to examine direct associations among study variables.

Finally, a chain mediation model was constructed using the lavaan package. Latent class membership served as the independent variable, family functioning and shame served as sequential mediators, and NSSI served as the dependent variable. The mediation models were estimated using maximum likelihood (ML) estimation. Sex, age, grade, and household income were included as covariates. Indirect effects were evaluated using 5,000 bootstrap resamples, and 95% percentile bootstrap confidence intervals were reported. In the hierarchical regression analysis, latent class membership was entered as a single ordinal variable coded as 1 = low-risk, 2 = moderate-risk, and 3 = high-risk. In the mediation analysis, latent class membership was dummy-coded, with Class 3 (high-risk) used as the reference category and Class 1 (low-risk) and Class 2 (moderate-risk) compared with the reference class. Sex, age, grade, and household income were included as covariates in the adjusted regression models.

## 3 Results

### 3.1 Participant characteristics

A total of 1,735 adolescents with left-behind experience were included in the study. The mean age was 14.76 ± 1.43 years. Among participants, 878 (50.6%) were male and 857 (49.4%) were female. Most participants were non-only children (85.4%), and annual household income was predominantly between RMB 20,000 and 50,000 (36.4%). More than half of participants (52.4%) experienced parental separation before the age of 6 years, and 59.3% belonged to the both-parent migration group. The mean scores for family functioning, shame, and NSSI were 13.52 ± 6.66, 22.78 ± 15.54, and 9.86 ± 7.10, respectively. Comparisons between adolescents with and without NSSI revealed significant differences in sex, age, grade, annual household income, age at parental separation, frequency of parent–child communication, attitudes toward parental migration, family functioning, and shame (all *p* < 0.05). Adolescents reporting NSSI were more likely to be female, younger, and have poorer family functioning and higher shame levels (see Table 1).

### 3.2 Latent class analysis of left-behind experience

LCA models containing one to six classes were estimated. Although model fit indices continued to improve as additional classes were added, the three-class solution demonstrated the optimal balance between statistical fit, classification accuracy, class size, and substantive interpretability. The three latent classes were characterized as follows: Class 1 (Low-risk left-behind group, 27.5%): later parental separation, shorter migration duration, and frequent parent–child communication. Class 2 (Moderate-risk left-behind group, 23.0%): intermediate migration duration and communication frequency. Class 3 (High-risk left-behind group, 49.6%): early parental separation, prolonged parental migration, both-parent migration, and infrequent communication. These findings indicate substantial heterogeneity in left-behind experiences among rural adolescents. The three-class solution was retained based on statistical fit, model parsimony, and substantive interpretability. From the two-class to the three-class solution, BIC decreased from 13713.15 to 13696.34 (ΔBIC = −16.81), and aBIC decreased from 13712.75 to 13695.95 (ΔaBIC = −16.80). In contrast, moving from the three-class to the four-class solution increased BIC and aBIC substantially, whereas the improvement in AIC was small. Although AIC decreased slightly through the five-class solution, additional classes increased model complexity and yielded less distinct and less readily interpretable subgroup patterns. The three-class solution therefore provided a more parsimonious and substantively interpretable representation of heterogeneity in left-behind experiences and was selected as the final model. The conditional item-response probabilities for the selected three-class solution are presented in Supplementary Table S1, providing the underlying probability estimates used to characterize and label the three latent classes (see Figure 1, Table 2).

**Figure 1:**
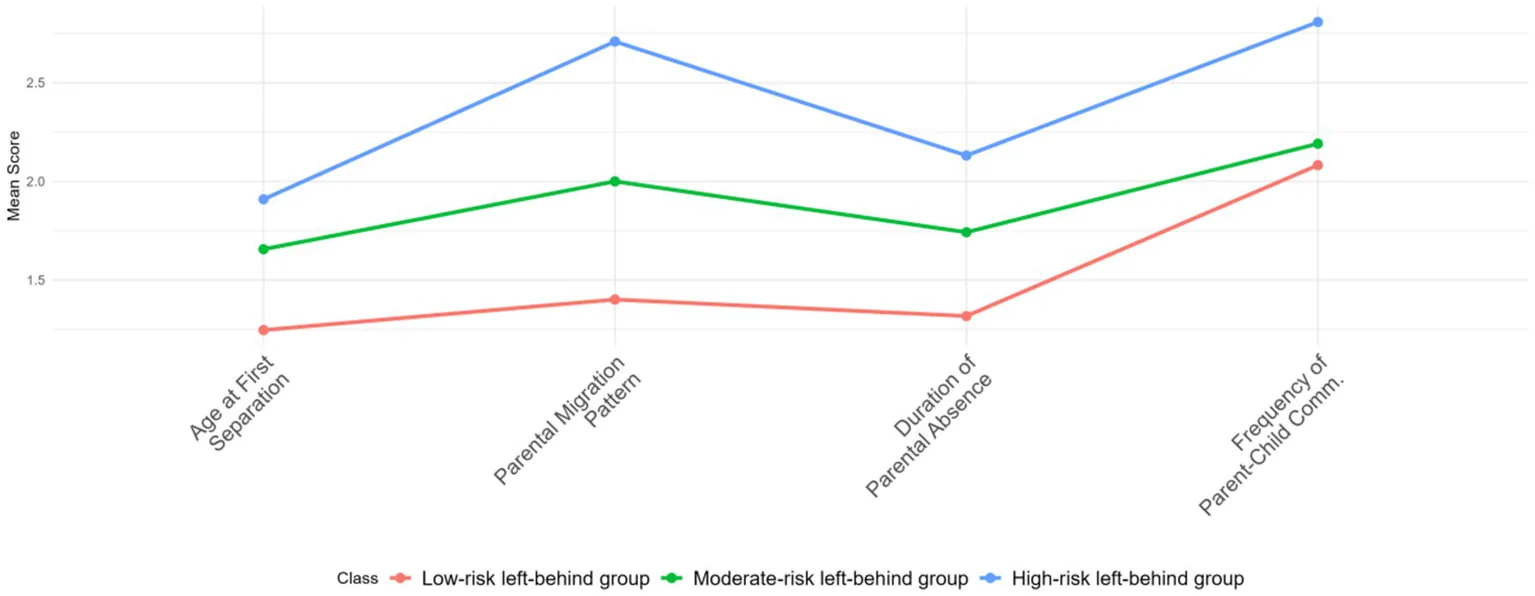
Conditional item-response probabilities for the three-class latent class model of left-behind experience among rural adolescents.

**Table 2:** Model fit indices for latent class analysis models with one to six classes.

Classes	AIC	BIC	aBIC	Entropy	Class proportions (%)
1	13693.39	13742.52	13742.12	—	100
2	13609.43	13713.15	13712.75	0.513	47.1/52.9
3	13538.04	13696.34	13695.95	0.72	27.5/23.0/49.6
4	13533.57	13746.47	13746.07	0.62	30.0/9.0/50.1/11.0
5	13530.39	13797.87	13797.47	0.606	6.0/9.6/29.6/7.6/47.1
6	13531.9	13853.97	13853.57	0.607	14.6/26.5/6.4/20.7/12.1/19.7

### 3.3 Correlation and multicollinearity analyses

Spearman’s rank correlation analyses demonstrated that latent class membership was negatively associated with family functioning (*ρ* = −0.149, *p* < 0.05) and positively associated with shame (*ρ* = 0.139, *p* < 0.001) and NSSI (*ρ* = 0.142, *p* < 0.05). Family functioning was negatively associated with shame (*r* = −0.132, *p* < 0.001) and NSSI (*r* = −0.235, *p* < 0.001). Shame was positively associated with NSSI (*r* = 0.256, *p* < 0.001). VIF values ranged from 1.019 to 1.036, indicating no evidence of problematic multicollinearity (see Table 3).

**Table 3:** Bivariate correlations among latent class membership, family functioning, shame, and NSSI.

Variable	Mean (SD)	1	2	3	4
1. Latent class	—	—			
2. Family functioning	13.52 (6.66)	−0.149*	—		
3. Shame	22.78 (15.54)	0.139***	−0.132***	—	
4. NSSI	9.86 (7.10)	0.142*	−0.235***	0.256***	—

Coefficients for correlations involving latent class (column 1) are spearman’s *ρ*, while coefficients among continuous variables are Pearson’s *r*. *p < 0.05; ***p < 0.001.

### 3.4 Hierarchical regression analyses

Latent class membership was significantly associated with family functioning (*β* = −0.049, *p* = 0.041). When shame was entered as the dependent variable, both latent class membership (*β* = 0.133, *p <* 0.001) and family functioning (*β* = −0.126, *p* < 0.001) were associated with shame. For NSSI, family functioning (*β* = −0.204, *p* < 0.001) and shame (*β* = 0.229, *p* < 0.001) remained significantly associated with NSSI. However, latent class membership was no longer significantly associated with NSSI (*β* = 0.000, *p* = 0.994) (see Table 4).

**Table 4:** Hierarchical regression analyses predicting family functioning, shame, and NSSI.

Predictor	Model 1 (family functioning)			Model 2 (shame)			Model 3 (NSSI)		
	*B*	*β*	*p*	*B*	*β*	*p*	*B*	*β*	*p*
Intercept	12.623	—	<0.001	32.433	—	<0.001	12.011	—	<0.001
Latent class	−0.39	−0.049	0.041	2.457	0.133	<0.001	0.005	0	0.994
Family functioning	—	—	—	−0.293	−0.126	<0.001	−0.832	−0.204	<0.001
Shame	—	—	—	—	—	—	0.399	0.229	<0.001
*R*	0.049			0.187			0.326		
*R* ^2^	0.002			0.035			0.106		
Adjusted *R*^2^	0.002			0.034			0.105		
*F*	4.199			31.505			68.671		

**Table 5:** Bootstrap estimates of direct, indirect, and chain mediation effects of family functioning and shame on the association between latent classes of left-behind experience and NSSI.

Pathway	*B*	Std. *β*	BootSE	95% CI	*p*
Direct effect (Class2 → NSSI)	−0.56	−0.06	0.74	−2.01, −0.49	0.013
Total indirect effect	−2.06	−0.076	0.73	−3.582, −0.708	0.005
Class2 → family functioning→ NSSI	−1.013	−0.037	0.45	−1.967, −0.196	0.024
Class2 → shame → NSSI	−0.904	−0.033	0.517	−2.014, 0.024	0.08
Class2 → family functioning→ shame → NSSI	−0.143	−0.005	0.072	−0.306, −0.027	0.017
Direct effect (Class1 → NSSI)	−0.16	−0.02	0.68	−1.48, −0.12	0.012
Total indirect effect	−2.81	−0.104	0.665	−4.181, −1.574	<0.001
Class1 → family functioning→ NSSI	−0.756	−0.028	0.357	−1.522, −0.129	0.034
Class1 → shame → NSSI	−1.947	−0.072	0.523	−3.071, −1.019	<0.001
Class1 → family functioning→ shame → NSSI	−0.107	−0.004	0.059	−0.243, −0.016	0.029

For each class comparison, the total indirect effect was calculated as the sum of the three specific indirect effects: (1) through family functioning, (2) through shame, and (3) sequentially through family functioning and shame.

### 3.5 Chain mediation analysis

Bootstrap mediation analyses were conducted using Class 3 as the reference group. Compared with the high-risk group, adolescents in the moderate-risk group exhibited a significant sequential indirect effect on NSSI through family functioning and shame (*B* = −0.143, 95% CI: −0.306, −0.027). The indirect effect through family functioning alone was statistically significant. In addition, the sequential pathway through family functioning and shame was significant, indicating that poorer family functioning was associated with higher levels of shame, which, in turn, were associated with greater NSSI severity. Similarly, adolescents in the low-risk group demonstrated a significant sequential indirect effect on NSSI (*B* = −0.107, 95% CI: −0.243, −0.016). The indirect effects through family functioning and shame were both statistically significant. Furthermore, the sequential pathway from latent class membership to family functioning, shame, and NSSI was supported because the bootstrap confidence interval excluded zero. After inclusion of family functioning and shame, the residual direct associations of both low-risk and moderate-risk class membership, relative to the high-risk reference class, with NSSI remained statistically significant (see Figure 2).

**Figure 2:**
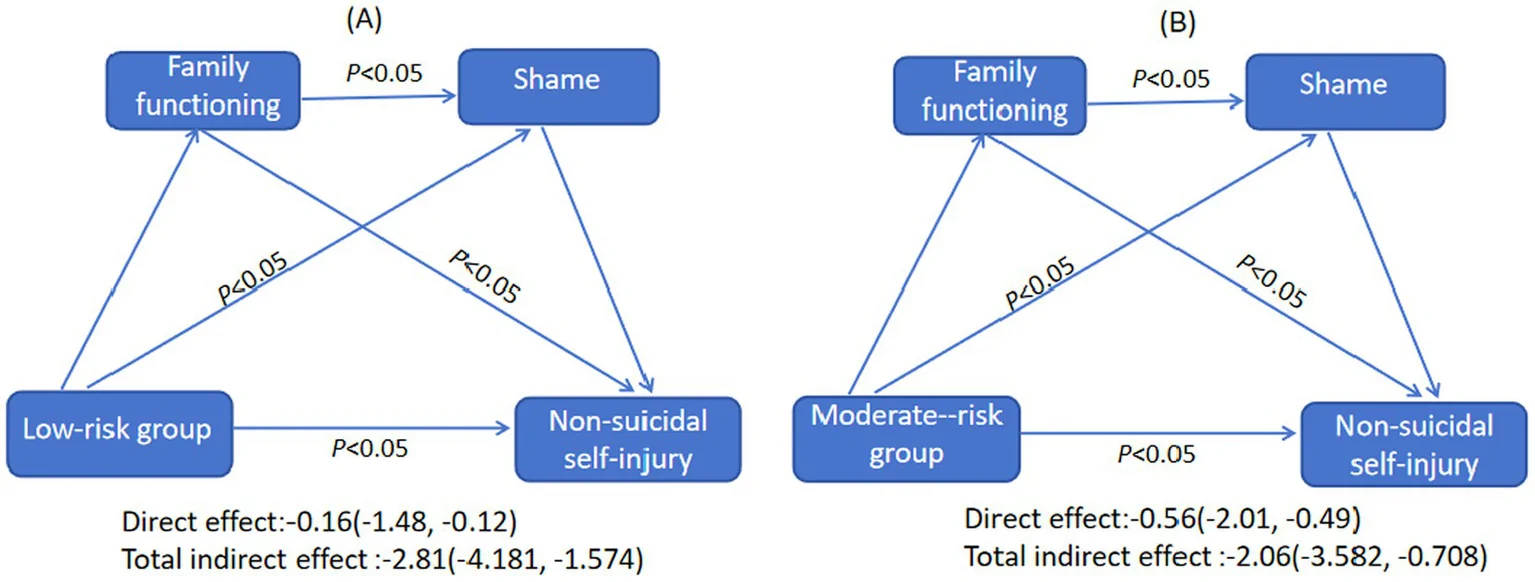
Sequential mediation model linking latent class membership, family functioning, shame, and non-suicidal self-injury. **(A)** Class1 vs. Class3, **(B)** Class2 vs. Class3. See Table 5 for detailed estimates of the direct and indirect effects and their 95% confidence intervals.

## 4 Discussion

The present study investigated the heterogeneity of left-behind experiences and their associations with non-suicidal self-injury (NSSI) among rural Chinese adolescents. Using latent class analysis (LCA), three distinct patterns of left-behind experience were identified. Furthermore, significant sequential indirect associations involving family functioning and shame were observed between left-behind experience and NSSI. These findings provide new evidence regarding the potential psychosocial processes associated with NSSI among left-behind adolescents and contribute to the growing literature emphasizing person-centered approaches to childhood adversity.

A major finding of this study was the identification of three distinct latent classes of left-behind experience. Specifically, adolescents could be categorized into low-risk, moderate-risk, and high-risk groups according to age at parental separation, migration duration, migration pattern, and frequency of communication with migrant parents. Previous studies have generally classified adolescents as either left-behind or non-left-behind, potentially overlooking substantial heterogeneity in migration-related experiences (21, 22). Consistent with person-centered developmental perspectives, the present findings suggest that left-behind adolescents are not a homogeneous population. Different combinations of parental migration characteristics may confer varying levels of psychosocial risk. The high-risk group was characterized by earlier parental separation, prolonged migration duration, both-parent migration, and infrequent parent–child communication. These characteristics have previously been associated with increased emotional and behavioral problems among left-behind children and adolescents (23, 24). Our findings extend previous research by demonstrating that these adverse migration-related characteristics cluster together and are associated with elevated NSSI risk. From the perspective of attachment theory, prolonged parental separation may undermine the development of secure attachment relationships and emotional regulation capacities (25). Adolescents in the high-risk group may therefore be particularly vulnerable to psychological distress and maladaptive coping behaviors, including NSSI.

Another important finding was the significant indirect association between left-behind experience and NSSI through family functioning. Family systems theory emphasizes that healthy family functioning provides emotional support, communication, adaptability, and psychological security for adolescents (26). Consistent with previous studies, better family functioning was associated with lower NSSI levels in the present study (27, 28). For left-behind adolescents, parental migration may reduce opportunities for emotional interaction and family communication, thereby weakening family cohesion and support. Consequently, adolescents experiencing poorer family functioning may demonstrate greater emotional dysregulation and vulnerability to self-injurious behaviors (27). Importantly, the mediation findings suggest that the association between left-behind experience and NSSI is not solely attributable to parental absence itself. Rather, the quality of family relationships and perceived family support may play a more important role in shaping adolescents’ psychological adjustment.

The present study also identified a significant indirect association between left-behind experience and NSSI through shame. Shame is a self-conscious emotion characterized by negative self-evaluation, self-criticism, feelings of inferiority, and social withdrawal (29). Previous studies have consistently demonstrated that elevated shame is strongly associated with NSSI and self-harming behaviors among adolescents (30, 31). Left-behind adolescents may be particularly susceptible to shame because parental absence may contribute to feelings of abandonment, self-blame, loneliness, and perceived inadequacy. These experiences may subsequently increase emotional distress and vulnerability to NSSI. Consistent with previous findings, adolescents reporting higher shame levels exhibited significantly greater NSSI severity. This result further supports theoretical models proposing that NSSI serves as a maladaptive strategy for regulating painful self-conscious emotions (30).

An important contribution of the present study is the identification of a significant sequential indirect association involving family functioning and shame. Specifically, the observed pattern is consistent with a pathway in which left-behind experience is associated with family functioning, family functioning is in turn associated with shame, and shame is associated with NSSI severity. These findings suggest that family functioning and shame may represent interconnected components of the association between left-behind experience and NSSI rather than independent processes. Adolescents experiencing dysfunctional family environments may receive insufficient emotional validation and support, thereby increasing vulnerability to negative self-perceptions and shame (32). Elevated shame may subsequently promote maladaptive emotion-regulation strategies, including NSSI (31, 33). These findings are consistent with developmental psychopathology frameworks emphasizing that adverse family experiences are associated with behavioral outcomes through multiple interacting psychological processes. The present chain mediation model therefore provides a more comprehensive explanation of how migration-related family adversity ultimately may be associated with self-injurious behaviors through interconnected psychosocial processes among adolescents. Nevertheless, because the present data are cross-sectional, the sequential mediation model should be interpreted as a statistical pattern of associations rather than evidence of a temporal or causal mechanism. Longitudinal studies are needed to determine the temporal ordering of these relationships.

The findings have several important implications for prevention and intervention. First, intervention programs should recognize the heterogeneity of left-behind experiences rather than treating all left-behind adolescents as a single population. Adolescents belonging to high-risk latent classes may require prioritized psychological monitoring and support. Second, family-based interventions should be strengthened. Improving parent–child communication, emotional support, and family cohesion may help reduce vulnerability to NSSI even when parental migration cannot be completely avoided. Third, shame-focused interventions may be beneficial. Cognitive-behavioral therapy, compassion-focused therapy, and self-compassion interventions have demonstrated effectiveness in reducing shame and improving emotional regulation (34). These approaches may therefore contribute to reducing NSSI risk among vulnerable adolescents.

Several limitations should be acknowledged. First, the cross-sectional design precludes causal inference, and longitudinal studies are needed to verify temporal relationships among left-behind experience, family functioning, shame, and NSSI. Second, all variables were measured using self-report questionnaires, which may introduce reporting bias. Third, participants were recruited from two rural schools in Hunan Province, limiting the generalizability of the findings. Fourth, although several associations were statistically significant, the effect sizes and explanatory power of the regression models were generally modest. The models explained 0.2%, 3.5%, and 10.6% of the variance in family functioning, shame, and NSSI, respectively, and most standardized regression coefficients were below |*β*| = 0.25. In particular, the model for family functioning explained only 0.2% of the variance, suggesting that the practical contribution of latent-class membership to family functioning may be limited. Given the relatively large sample size (*N* = 1,735), even small associations may reach statistical significance. Therefore, the findings should be interpreted in conjunction with effect sizes and explained variance rather than statistical significance alone, and latent-class membership should be regarded as one of multiple factors associated with these outcomes rather than as a strong independent predictor. Finally, although meaningful latent classes were identified, the moderate entropy value suggests that future studies should replicate these findings in larger and more diverse samples.

## 5 Conclusion

The present study identified three heterogeneous latent classes of left-behind experience among rural Chinese adolescents, including low-risk, moderate-risk, and high-risk groups. Membership in higher-risk left-behind classes was associated with poorer family functioning, higher levels of shame, and greater NSSI severity. The observed indirect associations were consistent with a sequential statistical pathway involving family functioning and shame in the relationship between left-behind experience and NSSI. These findings highlight the importance of considering heterogeneity in left-behind experiences and suggest that family functioning and shame may be relevant factors to consider in future NSSI prevention research among rural adolescents. Future longitudinal studies are needed to clarify the temporal ordering and potential mechanisms underlying these associations.

## Data Availability

The raw data supporting the conclusions of this article will be made available by the authors, without undue reservation.
