# Supplementary material for: Associations between latent classes of left-behind experience and non-suicidal self-injury among rural adolescents: the sequential mediating roles of family functioning and shame
Source: Front Public Health. 2026 Oct 14;14:1964648. doi: 10.3389/fpubh.2026.1964648 (PMC13713696; doi:10.3389/fpubh.2026.1964648)
Supplement: Supplementary file 1 [file Table_1.DOCX]

Supplementary Table S1. Conditional item-response probabilities for the three-class latent class model of left-behind experience

| Indicator | Response category | Class 1: Low-risk | Class 2: Moderate-risk | Class 3: High-risk |
| --- | --- | --- | --- | --- |
| **Age at first parental separation** | 0–6 years | 0.370 | 0.493 | **0.711** |
|  | 7–12 years | **0.432** | 0.385 | 0.203 |
|  | ≥13 years | 0.198 | 0.122 | 0.086 |
| **Parental migration pattern** | Father-only migration | 0.269 | **0.401** | 0.187 |
|  | Mother-only migration | **0.205** | 0.081 | 0.029 |
|  | Both-parent migration | 0.526 | 0.518 | **0.784** |
| **Duration of parental absence** | <1 year | 0.130 | **0.779** | 0.135 |
|  | 1–3 years | **0.823** | 0.000 | 0.237 |
|  | >3 years | 0.047 | 0.221 | **0.628** |
| **Frequency of parent–child communication** | Every 2 weeks | 0.510 | **0.782** | 0.530 |
|  | Every 3–4 weeks | **0.322** | 0.135 | 0.145 |
|  | Every 1–6 months | 0.116 | 0.057 | 0.121 |
|  | Less than once every 6 months | 0.053 | 0.026 | **0.205** |
